# Evaluation of a program to improve hand hygiene in Kenyan hospitals through production and promotion of alcohol-based Handrub – 2012-2014

**DOI:** 10.1186/s13756-018-0450-x

**Published:** 2019-01-03

**Authors:** Linus Ndegwa, Kelly M. Hatfield, Ronda Sinkowitz-Cochran, Emily D’Iorio, Neil Gupta, James Kimotho, Tiffanee Woodard, Sandra S. Chaves, Katherine Ellingson

**Affiliations:** 1 Influenza Program, US Centers for Disease Control and Prevention-Kenya, Nairobi, Kenya; 2 Division of Healthcare Quality Promotion, US Centers for Disease Control and Prevention, Atlanta, GA USA; 3 Production Unit, Kenya Medical Research Institute (KEMRI), Nairobi, Kenya; 4 Influenza Division, National Center for Immunization and Respiratory Disease, US Centers for Disease Control and Prevention, Atlanta, GA USA; 5 Department of Epidemiology and Biostatistics, The University of Arizona College of Public Health, Tucson, Arizona USA

**Keywords:** Hand hygiene, Alcohol-based handrub, Compliance

## Abstract

Although critical to prevent healthcare-associated infections, hand hygiene (HH) compliance is poor in resource-limited settings. In 2012, three Kenyan hospitals began onsite production of alcohol-based handrub (ABHR) and HH promotion. Our aim is to determine the impact of local production of ABHR on HH compliance and perceptions of ABHR.

We observed 25,738 HH compliance opportunities and conducted 15 baseline and post-intervention focus group discussions. Hand Hygiene compliance increased from 28% (baseline) to 38% (post-intervention, *p* = 0.0003). Healthcare workers liked the increased accessibility of ABHR, but disliked its smell, feel, and sporadic availability. Onsite production and promotion of ABHR resulted in modest HH improvement. Enhancing the quality of ABHR and addressing logistical barriers could improve program impact.

## Introduction

Healthcare-associated infections (HAIs) cause preventable illness and death in patients around the globe [1]. Hand hygiene (HH) by healthcare workers (HCWs) is critical to preventing HAIs, but healthcare facilities often fall short of HH compliance goals [2]. Interventions to improve HCW HH typically include education, reminders, feedback, administrative support, and access to alcohol-based handrub (ABHR) [3]. The Centers for Disease Control and Prevention (CDC) and the World Health Organization (WHO) recommend using ABHR for HH in healthcare settings, except in situations requiring the physical removal of microbes with soap and water (e.g., for pathogens *Clostridioidies difficile* or norovirus, or if hands are visibly soiled) [4, 5]. Evidence-based guidelines favor ABHR over soap and water in most cases because ABHR is more effective in killing most pathogens, takes less time to use, dries automatically, irritates hands less, and can be used at the patient bedside [6]. For facilities in low- and middle-income countries, commercially produced ABHR can be too expensive, although the components of ABHR are relatively cheap.

In 2009, the WHO published an implementation guide for HH improvement in hospitals worldwide. Included in the WHO toolkit were protocols for local production of ABHR, HH promotional materials, and tools for auditing HH compliance [4]. In 2011, the Kenya Ministry of Health and the CDC-Kenya adapted the WHO toolkit to train Kenyan pharmacists, HCWs, and other administrative staff in three hospitals on production of ABHR and on improving HH practices. The HH improvement program began in 2012. We sought to determine the effect of this program on HH compliance and perceptions of ABHR.

## Methods

### Participating hospitals

The Kenya Ministry of Health and CDC-Kenya invited three hospitals to participate in the HH improvement program, including a national hospital (Hospital A with 1800 beds), a regional referral hospital (Hospital B with 300 beds), and a district hospital (Hospital C, with 200 beds). Since 2009, the selected hospitals had been participating in an HAI surveillance program, which included hiring and training of surveillance officers to track HAIs on selected hospital wards [7]. Surveillance officers tracked HH compliance on their assigned HAI surveillance wards during baseline (December 2011 to May2012) and post-intervention periods (May or June 2012 to October 2014, including five wards from Hospital A, one from Hospital B, and one from Hospital C. Although measurement of HH compliance occurred in these select wards because of the surveillance officer capacity, the HH improvement intervention was implemented hospital-wide.

### Hand hygiene improvement program

**Fig. 1:**
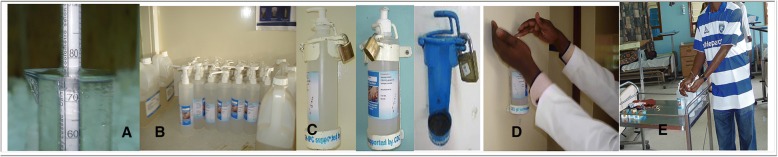
Photographs of local production of alcohol based hand rub (ABHR) and use within facilities: **a** verification of alcohol concentration using an alcoholmeter; **b**) quarantine of product for 72 h prior to dispensing to wards; **c**) mounting of ABHR bottles with custom holders, showing locks to prevent theft and rust; **d**) healthcare worker use of ABHR at the point of care; and E) placement of bottles on trollies for use during patient rounds

Each participating hospital selected three staff members — a nurse, a pharmacist, and a clinician champion — to attend a central training in October 2011 in Nairobi. To design the training, Kenya-based and US-based epidemiologists adapted training materials from the WHO Guide to Implementation of Hand Hygiene Improvement Programs. All participants were trained on ABHR production using validated WHO standards [4], and on HH promotion. Per the WHO toolkit for local production of ABHR, we trained participants to produce 10-l vats of ABHR, validate alcohol concentration, distribute to containers, and to quarantine for 72-h before dispensing to wards (Fig. 1). The HAI surveillance officers from participating hospitals received additional training on HH compliance auditing in accordance with the WHO Five Moments for Hand Hygiene [4].

Education on appropriate indications for HH and technique for use of ABHR was included in trainings for HCWs and incorporated into continuing medical education (CME) activities at each hospital. Posters adapted from WHO “Save Lives Clean Your Hands” promoting ABHR use were placed in visible locations. Placement of locally produced ABHR and posting of the promotional materials occurred in April 2012 for Hospital A and in June 2012 for Hospitals B and C.

Between June 2012 and October 2014, pharmacists at each hospital oversaw production of ABHR with a 75% alcohol concentration using 99.8% isopropyl alcohol, 6% hydrogen peroxide, and 99% glycerol. Bottles containing 500 mL of ABHR were mounted between patients’ beds using custom-made holders and at ward entrances. Pharmacists and clinician champions gave all HCWs small refillable bottles at the beginning of the intervention period and encouraged them to carry bottles in their pockets. Clinician champions also encouraged HCWs to place bottles on trollies used for patient rounds (Fig. 1). At hospital A, 166 wall mounts were installed and 167 HCWs were trained via CME; at Hospital B, 176 wall mounts were installed and 176 HCWs trained; and at Hospital C, 54 wall mounts were installed and 52 HCWs trained.

### Mixed methods evaluation

To evaluate implementation of the HH improvement program, we analyzed HH compliance and conducted focus group discussions before (baseline) and after the intervention (post-intervention). From December 2011 through October 2014, surveillance officers assessed HH compliance on their assigned wards at unannounced times at least twice a week; officers were instructed to observe HH opportunities for 20 min per audit and to use the WHO audit form. Healthcare workers were not told that they were being audited for HH compliance, although they knew about the HH improvement program and HAI surveillance.

Hand hygiene compliance was calculated for each hospital and ward during the baseline phase (December 2011 through April or May 2012) and for the follow-up period (May or June 2012 through October 2014) month post-intervention time period. Compliance was stratified by hospital, ward type, HCW type, and HH indication (Table 1). Since observations were non-independent, we assessed the statistical differences in HH compliance pre- and post- intervention using generalized estimating equation (GEE) models with a logit link and an adjustment for repeated measures for each of the seven wards evaluated.

**Table 1:** Baseline and post-intervention hand hygiene compliance, stratified by hospital, ward type, healthcare worker type, and indication. Odds ratios comparing post-intervention to baseline were calculated adjusting for repeated measures on each of the seven wards with complete reporting

	Pre-Intervention (Baseline)		Post-Intervention		Odds Ratio	
	Number of Opportunities Observed	Compliance	Number of Opportunities Observed	Compliance	(95% Confidence Interval)	*P* value
Overall	2809	28%	22,929	38%	*1.59 (1.24, 2.05)*	*0.0003*
Hospital						
Hospital A	1930	28%	16,675	39%	*1.65 (1.22, 2.24)*	*0.0012*
Hospital B	299	31%	1844	47%	*2.18 (2.05, 2.32)*	*< 0.0001*
Hospital C	580	27%	4410	31%	1.20 (0.62, 2.33)	0.5943
Ward Type						
ICU (1 unit)^a^	629	43%	4680	50%	*1.30 (1.10, 1.54)*	*0.0021*
Medical/ Surgical (1 ward)^a^	298	18%	2160	31%	*2.03 (1.49, 2.77)*	*< 0.0001*
Specialty (3 wards)	779	24%	8156	37%	1.75 (1.14, 2.71)	0.0113
Pediatrics (2 wards)	1103	24%	7933	34%	1.64 (0.99, 2.70)	0.0539
Healthcare Worker Type^b^						
Medical Officers	759	25%	4911	37%	*1.73 (1.19, 2.50)*	*0.0038*
Clinical Officers (Physician Assistant)	520	20%	3054	26%	1.54 (0.98, 2.43)	0.0611
Nurses	808	31%	7457	40%	1.43 (0.99, 2.05)	0.0562
Students	343	35%	4630	46%	*1.85 (1.18, 2.91)*	*0.0077*
Technicians	97	32%	276	34%	*1.45 (1.19, 1.77)*	*0.0002*
Others	282	31%	2590	34%	1.25 (0.79, 1.97)	0.3367
Indication^c^ (WHO “Moments”)						
Before touching patient	610	2%	5623	4%	*2.24 (1.24, 4.02)*	*0.0072*
Before clean/Aseptic task	457	1%	3786	5%	*14.4 (1.65, 125.74)*	*0.0159*
After body fluid exposure	30	93%	908	74%	0.16 (0.33, 0.99)	0.0479
After touching patient	1034	55%	9810	64%	*1.67 (1.13, 2.47)*	*0.0108*
After Patient Environmental Exposure	675	26%	4431	44%	1.44 (1.00, 2.09)	0.0516

^a^Did not adjust for repeated measures, only one unit compared

^b^Missing healthcare worker type for 11 observations in the post-intervention period

^c^Missing indication for 3 observations in the baseline period and 18 observations post-intervention

Italic entries are statistically significant

Focus group Discussions (FGDs). A trained moderator conducted separate FGDs by HCW type – clinicians, nurses, and support staff – using a standardized script. During the baseline period, 72 HCWs from the three hospitals participated in nine FGDs. Participants were asked about previous use of ABHR, and what they liked and disliked about ABHR based on any experience with the product. Trained moderators conducted the Post-intervention FGDs at least 1 year following implementation of the HH improvement program. Two hospitals participated in six post-intervention FGDs, and 32 HCWs were recruited. Participants were asked what they liked and disliked about the ABHR produced as part of the intervention and how the program could be improved. One hospital did not participate in post-intervention FGDs because it was experiencing a HCWs strike and was severely understaffed. Post-intervention FGD participants were not necessarily the same individuals who participated in baseline FGDs. All FGDs were recorded and transcribed. A research team read transcripts from each focus group prior to coding, which was performed according to standard qualitative “immersion” methodology [8]. The research team then created a list of codes to describe key themes and reviewed transcripts again to assign standardized themes.

Quantitative data analysis was conducted in SAS (V9.3, Cary, NC) and qualitative analysis was conducted using MAXQDA (Version 10, Amtsgericht Berlin Charlottenburg, Germany). The evaluation protocol was reviewed and approved by the KEMRI and CDC institutional review boards.

## Results

### Hand hygiene compliance

**Fig. 2:**
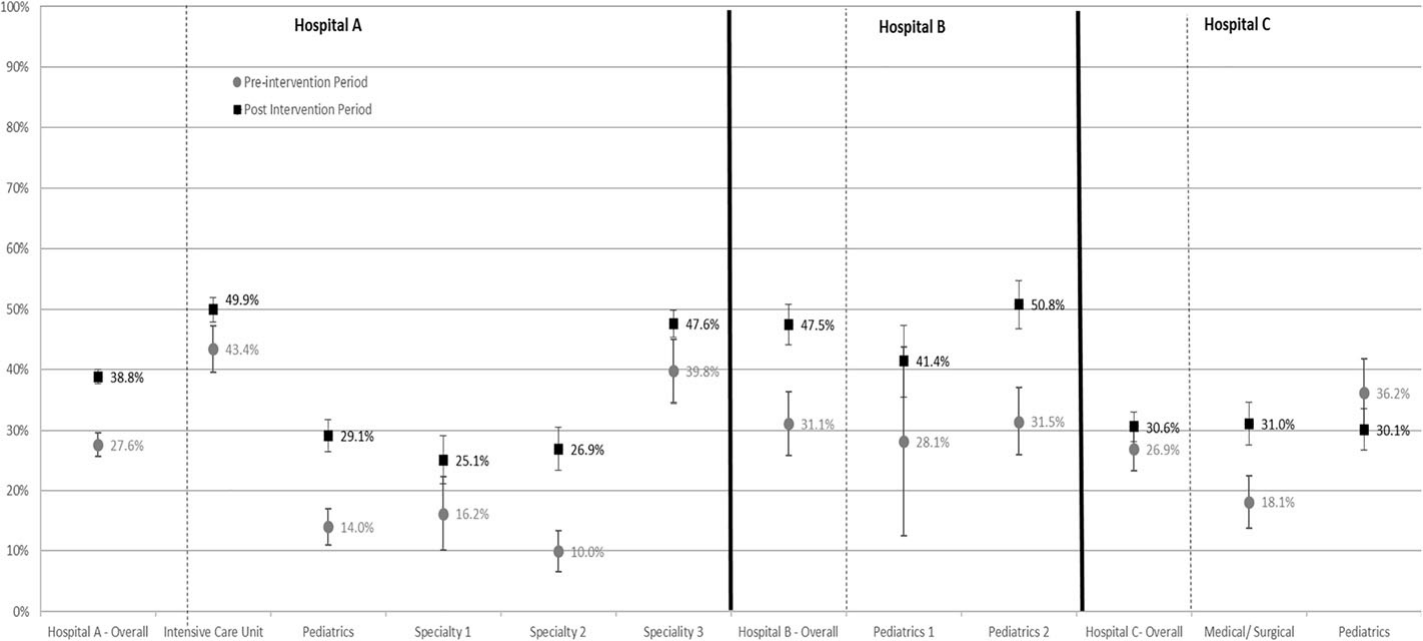
Hand hygiene compliance by hospital and ward type in the baseline phase (December 2011 through May/June 2012) and intervention phase (June/July 2012 – November 2014), for three hospitals in Kenya

Among the seven wards where HH compliance was measured, surveillance officers observed 2809 HH opportunities in the baseline period and 22,929 in the follow-up period. Overall, HH compliance increased from 28 to 38% (*p* = 0.0003) (Table 1). Two of the three hospitals demonstrated statistically significant improvement in HH compliance (Fig. 2). Improvements across all ward and HCW types were noted. During the baseline period, HH compliance was highest when the indication for HH was after HCW exposure to potential contaminants [i.e., after body fluid exposure (93%), after touching patient (55%), and after contact with patient’s surroundings (26%)] as opposed to before patient contact [i.e., before touching a patient (2%) and before a clean/aseptic task (1%)]. During the post-intervention period, compliance increased to 64% after touching a patient (*p* = 0.01), 5% before a clean/aseptic task (*p* = 0.02), and 4% before touching a patient (*p* = 0.007), and to 44% after exposure to patient environmental surroundings (*p* = 0.05). Compliance decreased after body fluid exposure from 93 to 74% (*p* = .048), although surveillance officers observed the fewest number of opportunities overall for this indication during the baseline period.

### Focus group discussions

At baseline, participants reported previous experience with commercially produced ABHR used outside the hospital or periodically donated to the hospital by an outside source. When asked what they liked about ABHR at baseline, HCWs’ responses most frequently elicited the themes of perceived efficacy of the product and convenience. For post-intervention FGDs respondents, when asked what they liked about ABHR, the most frequently elicited themes were convenience and availability. When asked what they disliked most about ABHR, baseline FGDs respondents emphasized the strong smell, residue, and dryness of commercially produced ABHR; post-intervention FGD respondents reported similar dislikes for locally-produced ABHR but favored commercially-produced ABHR, which had additives to reduce the harsh feel and smell.

The HCWs reported that having locally produced ABHR led to increased compliance: “It (having the handrub) saves time, you don’t have to look for a drier or take a trip to the sink. You just use it.” They also reported that the mounted dispensers created a cue to action: “seeing them is a reminder, it creates awareness.” When asked for input on the next steps for improving HH in the wards post- intervention, the most common themes elicited in baseline and post- intervention groups were education and performance feedback. Participants recommended promoting awareness among the public, the media, and caregivers, and creating a forum for consistent HAI compliance feedback to HCWs. Post-intervention respondents frequently discussed the theme of “improved packaging,” which included improving the container nozzle (to prevent leaking), fixing rusty padlocks, improving the labeling, and making the sanitizer containers more visually appealing.

## Discussion

The findings reported in this study – low baseline HH compliance and modest improvement with an ABHR production and HH promotion campaign – are consistent with similar studies from Ethiopia and Mali [9, 10]. When considering countries across the globe that have implemented similar programs, Kenyan hospitals had lower baseline HH rates, but demonstrated a similar magnitude of improvement with implementation of an improvement program following WHO protocols [11]. Consistent with the broader HH literature, HH compliance was highest after HCW exposure to a patient or the patient environment. The extremely low HH compliance before patient contact, even after the intervention, reflects a lack of understanding of the role of HH in patient safety. Educating HCWs, both in training and throughout their careers, about the role that HCWs hands play as a vehicle for disease transmission among patients could improve compliance before patient contact [2].

Focus group findings suggest that HCWs were receptive to ABHR production and HH improvement program, but also shed light on the program’s shortcomings. First, HH compliance feedback was given sporadically at ward meetings or during CME training sessions, but HCWs noted that systematic feedback diminished shortly after initiation of the intervention. Further, HCWs cited multiple problems with maintenance and packaging of the ABHR. After an initial rash of thefts of mounted bottles, program coordinators installed padlocks at the request of the hospitals. The locks rusted over time and keys were not always available when bottles needed refilling, which led to less consistency in availability of ABHR. Finally, the HCWs noted key differences between the locally-produced ABHR and commercial products: “The smell is too much; compared to the outside (commercial ABHR) products,” said one clinician. Another HCW added that commercial products had additives “like an oil so your hand remains soft”.

This evaluation was subject to a number of limitations. Although the ABHR was available for all wards at the participating hospitals, we only evaluated HH compliance on a subset of wards that were also targeted for HAI surveillance; therefore, findings may not be representative of the impact of the intervention in all wards. Furthermore, surveillance officers auditing HH opportunities worked mostly during weekdays, so night and weekend shifts were underrepresented. At Hospital C, there was a strike among HCWs during the intervention period, which led to gaps in care and HH observations on the hospital’s wards. Challenges associated with the strike illuminate the importance adequate staffing in achieving hand hygiene compliance and consistent implementation of the quality improvement activities necessary to maintain optimal compliance.

## Conclusions

This evaluation of an ABHR production and HH promotion program at three Kenyan hospitals demonstrated statistically significant but clinically modest improvement in HH compliance. Focus group findings suggest that logistical challenges – leaky pumps, rusty locks, and inconsistent refilling of mounted dispensers – lessened the impact of the program. Low rates of HH before patient contact suggest that clinical training and CME programs must emphasize the critical role that HCW hands can play in transmitting and preventing patient infections.

## Abbreviations

ABHR: Alcohol-based Handrub
CDC: Centers for Disease Control and Prevention
CDC-Kenya: Centers for Disease Control and Prevention-Kenya
CME: Continuing Medical Education
FGDs: Focus group Discussions
GEE: Generalized estimating equation models
HAIs: Healthcare-associated infections
HCWs: Healthcare Workers
HH: Hand Hygiene
KEMRI: Kenya Medical Research Institute
SAS: Statistical Analysis System
SERU: Scientific & Ethics Review Unit
WHO: World Health Organization
